# Active tuberculosis and multiple sclerosis: the importance of screening before treatment

**DOI:** 10.1055/s-0045-1810406

**Published:** 2025-08-04

**Authors:** Leizian de Souza Amorim, Paloma Peter Travassos Zaidan, Felipe Toscano Lins de Menezes, Enedina Maria Lobato de Oliveira

**Affiliations:** 1 Universidade Federal de São Paulo, Escola Paulista de Medicina, Departamento de Neurologia e Neurocirurgia, São Paulo SP, Brazil.

**Keywords:** Multiple Sclerosis, Tuberculosis, Risk Assessment

## Abstract

Tuberculosis (TB), a chronic infection caused by the *Mycobacterium tuberculosis* complex, has an increased risk of reactivation in conditions that affect the immune system, such as MS, and its treatment with disease-modifying drugs (DMDs). The present is a retrospective study of 2,036 patients diagnosed with MS followed at the Department of Neurology and Neurosurgery of Escola Paulista de Medicina, Universidade Federal de São Paulo, from February 1994 to September 2023. Of that total, 6 were included in this case series, taking different DMDs: fingolimod (n = 2), interferon beta 1a (n = 2), glatiramer acetate (n = 1) and cyclophosphamide (n = 1). In our study, two patients experienced worsening disability during tuberculosis treatment, while three others had increased disability after completing treatment. We reinforce the importance of screening all patients eligible for DMD treatment, especially the highly effective modern ones, and the importance of developing research-based guidelines for screening infectious diseases among patients with MS.

## INTRODUCTION

Tuberculosis (TB) is a chronic infection caused by the *Mycobacterium tuberculosis* complex, known as Koch's bacillus, an alcohol-acid-resistant and obligate aerobic bacillus. It remains a serious public health problem worldwide. 1 2 According to the World Health Organization (WHO), one-third of the world's population is infected by *M. tuberculosis* , but only 10% develop the active form of TB during their lifetime. 3 Brazil has the highest prevalence rate in Latin America, accounting for 33% of cases, followed by Peru (13.1%) and Mexico (10.7%). 4 Fatigue is the most prevalent TB symptom in multiple sclerosis (MS) and requires cautious attention. 5

This disease is transmitted by air, in which the inhaled bacilli reach the lung tissue. The infection and progression to the active clinical form depend on multiple causes resulting from environmental, social, and genetic factors. Only a few genetic variants have been identified, shedding light on human's susceptibility to TB. 6 These genes play a role in interferon responses, indicating that chronic activation of the peripheral immune system occurs before the onset of active disease. 6 Furthermore, resistance to TB has been observed to follow a complex, non-Mendelian, inheritance pattern. 6

A cell-mediated immune response is formed, followed by the development of delayed-type hypersensitivity with impaired adaptive immunity. 7 Furthermore, *M. tuberculosis* infection may result in initial control, active disease, or latent TB. Conditions that affect the immune system, such as MS and its treatment with disease-modifying drugs (DMDs), can increase the risk of infections, including TB. The active subtype in adults usually occurs by reactivating latent foci previously under immunological control. In MS, reactivation is a consequence of DMD treatment, and the inflammatory response caused by *M. tuberculosis* can worsen MS activity. 8 We aimed to describe a case series of patients with MS and TB.

## METHODS

We performed a retrospective observational study based on patient medical records from the Neuroimmunology Clinic of Hospital São Paulo, representing part of Brazil's public health system. The study was approved by the Universidade Federal de São Paulo (UNIFESP) Ethics Committee (CAAE: 40467720.8.1001.5505).

There were 2,036 patients followed at UNIFESP's Neuroimmunology Clinic selected for inclusion if admitted between February 1, 1994, and June 30, 2019. Among them, we retrieved 811 people with MS, according to the current diagnostic criteria (2017 and revisions of McDonald criteria). The study was based on ambulatory patients who did not require an in-day hospital stay. Those who were 18-years-old or younger at the time of their first appointment and those who had fewer than three visits or less than 6 months of follow-up were not included, leaving 629 participants.

**Figure 1:**
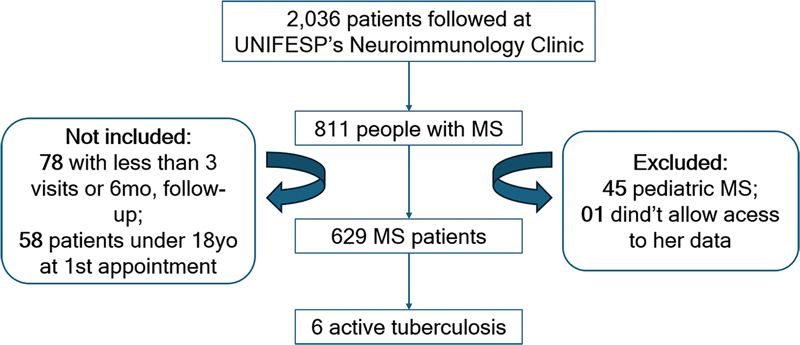
Abbreviations: mo., months; MS, multiple sclerosis; UNIFESP, Universidade Federal de São Paulo; yo, years old. Flowchart showing patient selection with inclusion and exclusion criteria.

Patients were not regularly screened for TB; however, in 95 of them, either the Purified Protein Derivative (PPD) or the interferon gamma release assay (IGRA) tests were performed due to a treatment switch. All charts were reviewed, and we identified six individuals with active TB during follow-up, the topic of this study. This condition was confirmed through the positive tests and complementary studies, such as biopsy, chest X-rays, or bronchoalveolar lavage via bronchoscopy ( Figure 1 ).

**Figure 2:**
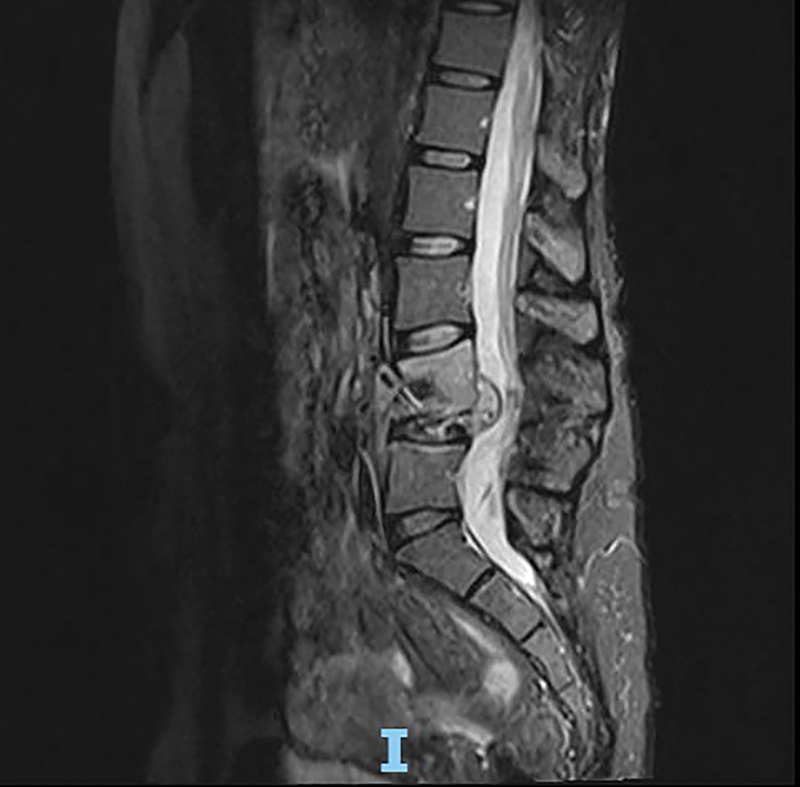
Sagittal short tau inversion recovery (STIR) magnetic resonance imaging (MRI) sequence of the lumbar spine, showing signs of total collapse of the L4 vertebral body with foraminal compression and spinal cord sclerosis in patient 1.

**Figure 3:**
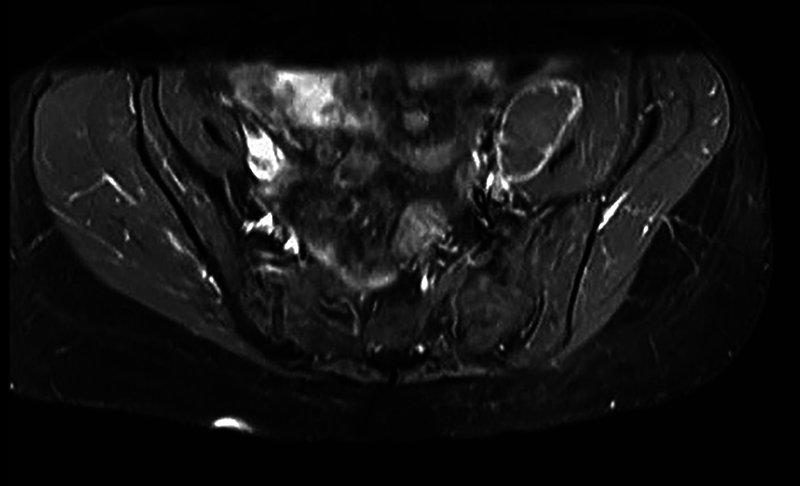
Axial T2-weighted magnetic resonance imaging (MRI) sequence of the lumbar spine, showing retroperitoneal abscess as a tuberculosis complication in patient 1.

### Case series

This case series included patients who developed active tuberculosis during disease-modifying treatment (DMT) for MS. Table 1 summarizes the cases' demographics, diagnostic data, and particularities. Figures 1 and 2 ilustrate the MRI of patient 1, the most severe case.

**Table 1:** Patient characteristics

Characteristics	Case 1	Case 2	Case 3	Case 4	Case 5	Case 6
Gender	Female	Female	Female	Female	Male	Female
Age (years)*	40	37	40	61	39	54
MS subtype	SPMS	RRMS	RRMS	RRMS	SPMS	SPMS
MS diagnosis	01/04/2006	1/16/2018	01/08/2007	12/15/2008	5/20/2001	3/15/2001
Date of DMD initiation	1/30/2015	2/19/2019	01/12/2011	01/04/2015	8/21/2002	01/01/2001
TB diagnosis	09/01/2022	9/13/2019	07/07/2016	06/01/2019	03/04/2007	01/08/2004
DMD in use	Fingolimod	Fingolimod	Interferon beta 1a	Glatiramer	Cyclophosphamide	Interferon beta 1a
Time between DMD initiation and TB diagnosis (days)	2,618	155	1,492	1,496	1,665	1,308
Suspended DMD	07/01/2022	9/24/2019	05/10/2016	06/01/2019	03/04/2007	12/21/2004
DMD switch after TB	Yes, to natalizumab	No	Yes, to dimethyl fumarate	No	No	No
PPD	Not available	Not available	Positive	Not available	Not available	Positive
Lymphopenia(grades 1–4)**	1	2	0	0	0	0
TB clinical form	Disseminated	Disseminated	Ganglionic	Pulmonary	Ganglionic	Ganglionic
Symptoms	Progressive worsening of gait and chronic low back pain, L3-L4 fracture	Pain in the right clavicular region with progressive bone swelling and abscess formation	Acute febrile syndrome and pleuritic pain.	Asymptomatic	Supraclavicular lymph node enlargement	Cervical lymph node enlargement
TB localization	Right upper lobe	Colar bone and clavicular lymph node	Lungs	Lungs	Supraclavicular lymph nodes	Cervical lymph nodes
EDSS score a year before TB	6.0	1.5	1.5	2.5	6.5	1.0
EDSS during TB	6.5	1.5	1.5	2.5	6.5	1.0
EDSS score a year after TB	6.5	1.5	6.0	2.5	6.5	1.0
EDSS score in 2023	6.5	3.5	3.5	6.0	6.5	4.0
Annualized relapse rate	0.33	0.66	0.58	0.25	0.52	0.04
Time of last relapse before TB diagnosis (years)	8	1	5	4	1	4
New relapse a year after TB	No	No	Yes, 1	No	No	No
Need for hospitalization	Yes	Yes	Yes	No	No	No
New T2 or Gd+ lesions on MRI	2	0	1	0	0	0

Abbreviations: DMD, disease-modifying drug; EDSS, Expanded Disability Status Scale; Gd + , gadolinium-enhancing; MRI, magnetic resonance imaging; MS, multiple sclerosis; PPD, pulse dye densitometry; SPMS, secondary progressive multiple sclerosis; RRMS, relapsing-remitting multiple sclerosis; TB, tuberculosis.

Notes: *Actual age. **Lymphopenia grade 1– > 800; grade 2–800–500; grade 3–500–200; and grade 4– < 200).

Most patients were female, with a male to female rate of 1:5. None of them had an additional risk factor (diabetes, smoking, cancer, and living with contacts) for TB besides residing in Brazil, where the incidence is 45 per 100 thousand people according to the WHO database. All patients underwent a joint evaluation by the pulmonology team since, perhaps due to chronic immunomodulation, they presented atypical symptoms, thus requiring tissue-driven biopsy, and the biopsy showed the caseous granuloma in all cases. They received specific TB treatment for 6 to 12 months according to the current guidelines, which vary depending on the type.

The median therapeutic inertia for the TB treatment was 45 days, with DMT being withdrawn during the initial phase. There were two patients who required an MS treatment switch due to worsening of the disease, represented by worsening of the score on the Expanded Disability Status Scale (EDSS) or new relapses, detailed in Table 1 .

## DISCUSSION

We presented a case series of TB reactivation in patients taking different DMDs: fingolimod (n = 2), interferon beta 1a (n = 2), glatiramer acetate (n = 1), and cyclophosphamide (n = 1). The relationship between MS and TB is complex and can be divided into two axes. First, there is the risk of reactivation during DMD treatment ( Table 2 ) 16 17 18 19 20 . Second, the host's inflammatory response can be intense and lead to the development of disease activity. 7

**Table 2:** Relationship between tuberculosis and DMD for MS

Reference	Patients (n)	Patients with TB diagnosis	TB subtype	DMD	Country of study and incidence (per 100,thousand people)*	Title
Comi, 2015	3,044	2	Active	Teriflunomide	Multicentric	Pooled safety and tolerability data from four placebo-controlled teriflunomide studies and extensions
Dahdaleh, 2012	170	2	Active	Natalizumab	Ireland (7), Turkey (15)	Breathlessness, night sweats, and weight loss on natalizumab
Boyko, 2015	100	1	Active	Natalizumab	Russia (46)	A prospective, open, nonrandomized study on the safety and efficacy of natalizumab (tisabri) in the Russian population of patients with relapsing-remitting multiple sclerosis
Ferro, 2021	149	40	Latent	Undisclosed	Portugal (16)	Infectious Risk Mitigation in Patients with Multiple Sclerosis under Disease-Modifying Therapies – the Experience of a Collaborative Neurology-Infectious Diseases Approach
Cohen, 2012	376	1	Active	Alentuzumab	Multicentric	Alemtuzumab versus interferon beta 1a as first-line treatment for patients with relapsing-remitting multiple sclerosis: a randomized controlled phase-3 trial

Abbreviations: DMD, disease-modifying drug; MS, multiple sclerosis; TB, tuberculosis.

Notes: PUBMED search in July 2023: [tuberculosis] AND [interferon beta] AND [multiple sclerosis]; [tuberculosis] AND [teriflunamide]; [tuberculosis] AND [dimethyl fumarate]; [tuberculosis] AND [cladribine]; [tuberculosis] AND [fingolimod]; [tuberculosis] AND [natalizumab]; [tuberculosis] AND [alentuzumab]; [tuberculosis] AND [ocrelizumab]. Case reports were excluded. *According to World Health Organization data.

The investigation for TB should include clinical history, PPD or IGRA tests, and chest X-rays performed on every patient who received any second-line DMT or above. Bronchoalveolar lavage via bronchoscopy was performed on patients with high clinical suspicion of pulmonary TB who were unable to expectorate or had negative sputum smear and culture. Although TB screening was not routinely performed in the past—leading us to include only active cases in this study—we believe that all patients considered for oral or intravenous DMDs should undergo it. This study prompted a change in our protocol, which has since been implemented for all patients receiving oral or intravenous treatment for MS.

Since this study is based on a chart review, most patients treated before 2020, particularly those on first-line DMTs, did not undergo TB screening. However, every patient who transitioned to a second-line DMT after 2020 was screened. Second-line DMTs were classified according to the Brazilian Therapeutic Protocol for MS, which includes fingolimod, dimethyl fumarate, natalizumab, cladribine, and alemtuzumab.

Tuberculin and IGRA are recommended tests for latent TB. The tuberculin skin test has a sensitivity and specificity of 75%, and a negative result does not exclude the diagnosis, which is why the WHO guidelines ^5^ recommend IGRA to detect latent TB. This test measures the T-cell response stimulated by *M. tuberculosis* antigens. 9 However, DMTs and lymphocyte count can affect the results.

In a short report, 1,058 patients with MS were screened using IGRA, 59,4% (628) were on disease-modifying drugs. 10 Furthermore, 2% (21) were positive for TB, and 6.1% (65) were indeterminate. 10 Results were significantly different between DMTs: dimethyl fumarate (26,2%, n = 17) and fingolimod (29,2%, n = 19) had the highest incidence of indeterminate IGRA, suggesting a higher risk of indeterminate result if the therapy affects lymphocyte. 10 Furthermore, lymphopenia grade 3 or worse (OR: 9.39) and recent use of methylprednisolone also impact IGRA results (OR: 3.2). 11

Active TB in adults can result from the reactivation of latent foci. The medications used for MS act on cell-mediated immunity, most likely associated with disease activation or progression. 12 For instance, fingolimod, a modulator of sphingosine-1-phosphate receptors, may trigger TB reactivation by sequestering lymphocytes in lymphoid tissue. During fingolimod treatment, we observed two cases of TB. One patient had mild lymphopenia with prolonged exposure, while the other exhibited moderate lymphopenia with shorter medication exposure. During the clinical development program of cladribine tablets for MS, a total of three cases of TB were reported, all of which occurred in regions where it is endemic. One case of TB was fatal, and two resolved with treatment. 13

Treatment for MS does not rely on targeting humoral immunity. The anti-CD20 therapy, for instance, targets B-cells and impacts their function as antigen-presenting cells, thereby decreasing cell-based immunity. Reactivation risk is high in immunocompromised patients and moderated in people who received corticosteroids of more than 15mg/day for at least one month. 14

Brazil has a high prevalence of TB, and therefore, we are vaccinated at birth. This measure may impact the incidence of TB among Brazilian patients with MS. As such, we cannot assert that the prevalence of TB in this population is the same as that estimated for patients in other countries, which is at around 4.95%. 5 We believe it is essential to maintain infectious disease surveillance among patients treated with high-potency medications. Therefore, a specific history, physical examination, and chest x-ray are necessary to rule out active infection before treatment for MS.

In a recent Italian study with 174 patients, 19 tested positive for QuantiFERON-TB Gold, of which two patients had active disease, highlighting the importance of screening in patients with MS before treatment. 15 The PPD skin and IGRA tests should be done before starting treatment. A chest x-ray may also be suggested for patients from high-incidence countries or with TB symptoms.

All patients with MS candidates for immunomodulatory treatment should be tested for latent TB. The PPD and IGRA tests are readily available in Brazil, and patients should be evaluated and treated, if necessary, when starting treatment or switching medications. Treatment should follow the National Tuberculosis Prevention Program, Monitoring, and Control standard regimen. 8 The decision to quit the DMD or treat both conditions simultaneously varies between centers. Our center's decision is based on the patient's clinical status, MS activity, and the current DMDs. In the case of active TB infection, upon diagnosis, we advise pausing the current MS treatment and starting antibiotics for a minimum of 3 months before resuming. Hepatotoxicity risk should also be considered for combined treatment (TB and MS).

The present study reinforces the importance of screening all patients eligible for DMD treatment, especially the highly effective modern ones, and the importance of developing research-based guidelines for screening infectious diseases among patients with MS.
